# Mapping the Social Context of Type 2 Diabetes Management: A Network Analysis of Disease-Related Complications Among Black/African American Men

**DOI:** 10.1007/s40615-025-02644-x

**Published:** 2025-09-17

**Authors:** Tyler Prochnow, Parinika T. Joshi, Megan S. Patterson, Jeong-Hui Park, Matthew Lee Smith, Ledric D. Sherman

**Affiliations:** 1 School of Public Health, Texas A&M Health Science Center, 212 Adriance Lab Rd, College Station, TX USA; 2 Center for Health Equity and Evaluation Research, Texas A&M University, College Station, TX 77843 USA; 3 Center for Community Health and Aging, Texas A&M University, College Station, TX 77843 USA

**Keywords:** Type 2 diabetes, Social networks, Health disparities, Black/African American men, Social support, Chronic disease management

## Abstract

Social networks play a crucial role in type 2 diabetes (T2D) management, yet limited research examines these dynamics among Black/African American men who face disproportionate T2D burden. This study investigated associations between social network characteristics and negative T2D outcomes among 1,225 Black/African American men with T2D. Participants completed an online survey assessing their social networks and T2D outcomes including low blood sugar reactions, high blood sugar symptoms, and ketone presence. Multiple linear regression analyses revealed that while frequent diabetes-specific discussions were associated with more negative outcomes (β = .329, p < .001), general communication frequency (β = -.221, p < .001) and having very supportive network members (β = -.209, p < .001) demonstrated negative associations. Higher proportions of family members, including siblings (β = .089, p = .034), extended family (β = .105, p = .012), and children (β = .087, p = .015), in networks were associated with increased negative outcomes. Physical activity levels within networks showed significant negative association (β = -.093, p = .008), while perceived healthy eating behaviors showed no significant associations. These findings suggest the complex nature of social support in T2D management, where the quality of support may be more influential than quantity, and where family relationships may introduce competing demands that complicate disease management. Results emphasize the importance of considering both communication patterns and relationship dynamics in developing culturally tailored interventions for Black/African American men with T2D. Future research should examine how these social network characteristics evolve over time and influence long-term T2D outcomes.

## Introduction

Diabetes is among the top 10 causes of morbidity and mortality with a current global prevalence rate of 6.1% [1]. The International Diabetes Federation (IDF) states that the number of individuals with diabetes grew by 10% between 2014 and 2019, reaching 463 million globally [1]. Type 2 diabetes (T2D) is the most common form of diabetes, with approximately 34.2 million people in the United States (US) having the condition [2]. Over the past ten years, the prevalence of T2D has risen significantly, indicating an international health problem [1]. It is anticipated that the total number of T2D cases internationally will rise substantially, increasing from a projected 529 million cases in 2023 to 1.3 billion cases by 2050 [1].

Black/African Americans experience a disproportionate impact from T2D, exhibiting a prevalence rate of 17.4% compared to 13.6% among non-Hispanic White Americans [3]. More specifically, Black/African American men also have a higher prevalence of T2D (11.5%) as compared to White non-Hispanic men (7.7%)[3]. Black/African Americans are up to 77% more likely to develop T2D relative to non-Hispanic White Americans [4]. Further, the health consequences of unmanaged T2D include diabetic retinopathy, kidney failures, and diabetic foot [5]. Black/African Americans are almost 50% more likely to develop diabetic retinopathy than non-Hispanic whites and are 2.6 times more likely to develop end-stage renal disease due to diabetes compared to non-Hispanic white Americans [5]. An intersectional approach, as highlighted by the National Institute on Minority Health and Health Disparities (NIMHD), is essential when considering the multiple factors that influence T2D outcomes in minority men. This approach recognizes the intersection of race, gender, socioeconomic status, and other social identities in shaping health experiences and outcomes [6].

Men, especially those from minority groups, may face unique challenges to T2D management [7]. These challenges are influenced by a complex interplay of factors, including health beliefs, patient-provider communication, and social and structural barriers [8]. The Centers for Disease Control and Prevention (CDC) states that men are less likely than women to visit their doctor for regular checkups, eventually leading to delayed diagnosis and treatment of T2D [9]. Further, societal norms and expectations of masculinity may decrease help seeking behavior and social support which impacts T2D outcomes [10]. Women often report more emotional and social support compared to their male counterparts while less social support is related to an increased risk of diabetes complications [11, 12]. This social support can be a vital way to buffer stress which in turn can affect metabolic function and may lead to T2D [13].

Social network theory offers valuable insights into health behaviors and T2D management. The social contagion or behavioral hypothesis reports that healthy or unhealthy behaviors may also be acquired by people in the immediate social network [14]. Understanding the role of social networks that influence health behaviors and access to resources will be crucial for addressing health equity in T2D management. According to research, social networks have a significant effect on self-care during diabetes by influencing factors like emotional support, medication adherence, health literacy, and information dissemination [15]. Smaller network size is associated with increased T2D risk, and this holds with the fact that both sexes with T2D have smaller networks when compared to those having normal glucose metabolism [12]. Factors such as network size, diversity, and the types of support provided by network members can influence treatment adherence, lifestyle modifications, and overall health outcomes [16]. A more comprehensive knowledge of the social factors affecting Black/African American men's self-management behaviors can be acquired by understanding social network analysis in T2D research. Social network analysis places emphasis on the importance of social connection and social structure to determine the impact these connections may have on health and health behavior [17]. In this manner, this approach asks individuals to specifically think about people in their lives instead of broader feelings of support [17]. Further, this approach is able to determine specific aspects about these networks in detail that might be associated with T2D outcomes [17].

This study aims to investigate the role of social networks in the frequency of poor T2D outcomes (i.e., low blood sugar reactions, high blood sugar symptoms, and the presence of ketones) among Black/African American men. Further, by utilizing a conceptual framework that considers social network theory with cultural considerations that are relevant to this population, we hope to better understand the social dynamics surrounding the management of T2D. The primary objectives of this research are to examine the structure and composition of social networks among Black/African American men with T2D and investigate the relationship between social network characteristics and T2D self-reported outcomes. By addressing these objectives, the study aims to contribute to the development of more effective, culturally tailored programs for T2D management among Black/African American men.

## Methods

### Study Design

This cross-sectional study analyzed data collected through an online Qualtrics survey between February and June 2024. The survey examined social networks and T2D outcomes among Black/African American men, with recruitment facilitated through Cloud Research to enable targeted recruitment of this specific population. This methodology aligns with previous research demonstrating the effectiveness of online panels in reaching underserved populations [18].

### Participants and Procedures

The study included 1,225 Black/African American men diagnosed with T2D. Eligibility criteria specified that participants must: self-identify as Black/African American, identify as male, be at least 21 years old, self-report a T2D diagnosis, and reside in the US. Participants accessed the survey through a Qualtrics link after reviewing an Institutional Review Board (IRB)-approved information sheet ([anonymous]). Participants were guaranteed anonymity, and informed consent was obtained from all individuals prior to their involvement in the study. To ensure data quality, three attention checks were embedded within the survey following recommendations [19], and only those who passed all three were included in the final analysis.

### Measures

#### Social Networks (Independent Variables)

A multiple name generator approach was used to elicit participants' social networks [20, 21]. This comprehensive method allows for a detailed assessment of participants' personal networks (egocentric networks) related to their T2D management. Participants were asked to indicate people who: give them advice, they confide in, provide practical support, and make managing their T2D difficult. These responses resulted in a comprehensive list of social network members. Participants could list the same person across multiple prompts when applicable. For each person reported in their network, participants were asked to specify their relationship type (spouse, child, parent, friend, sibling, extended family member, healthcare provider, coworker, roommate, neighbor, or other). Participants also indicate whether each network member has T2D themselves (yes, no, I don't know). Health behaviors of network members are assessed through two key measures: perceived physical activity frequency and healthy eating habits. Both of these items were rated on a four-point scale (never, rarely, sometimes, often). Perceived supportiveness specific to diabetes management was evaluated using a four-point scale (not at all supportive, a little supportive, sometimes supportive, very supportive). Contact frequency with each network member was measured through two questions using a six-point scale ranging from several times daily to never. First, participants were asked how frequently they talked to each specific network member. Then participants were asked how frequently they talked to that member about T2D specifically.

Responses for all network members were aggregated to create multiple network-level variables, including network size, proportion of network members by relationship type (e.g., spouse, child, parent, friend, other family member, health care provider), percentage of network members with T2D, relationship heterogeneity (measure of how many different relationships showed up in their network), mean communication frequency, frequency of diabetes-specific discussions, average level of network support, percent of very supportive members, and perception of members’ health behaviors (eating healthy and being physically active). Due to the compositional nature of network relationship type data (percentages summing to 100%), centered log-ratio transformations were performed on network composition variables prior to analysis. This transformation addresses the constraints and dependencies inherent in compositional data while preserving the relative relationship information.

#### Negative T2D Outcomes (Dependent Variables)

We assessed adverse T2D outcomes using four items from the Diabetes Care Profile (DCP) [22, 23]. These validated measures assessed the frequency of: low blood sugar reactions with symptoms (sweating, weakness, anxiety, trembling, hunger, headache) in the past month; high blood sugar symptoms (thirst, dry mouth/skin, increased urinary sugar, reduced appetite, nausea, fatigue) in the past month; ketones in urine in the past month; and severe low blood sugar reactions requiring assistance in the past year. Following the DCP scoring protocol, participants rated each item on a 5-point scale (0 = 0 times, 1 = 1–3 times, 2 = 4–6 times, 3 = 7–12 times, 4 = more than 12 times). We analyzed individual items and a composite score calculated by summing all four items (possible range 0–16), an approach validated in previous diabetes research (Cronbach’s α = 0.86) [22].

#### Demographic Variables

The analysis included demographic controls: age, residential area (rural, suburban, urban, other), educational attainment (Some high school, no diploma; High school diploma/GED; Some college, no degree; Technical/vocational training; Associates degree; Bachelor's degree; graduate degree), employment status (student, employed, unemployed, retired, unable to work), annual household income (in $25,000 increments), marital status (married/partnered, never married, divorced/separated, widowed), and Body Mass Index (BMI).

### Data Analysis

The characteristics of the participants were summarized using descriptive statistics, which included the calculation of frequencies, means, and standard deviations. We conducted multiple linear regression analyses to examine associations between social network characteristics and negative T2D outcomes, following analytical approaches established in previous social network research [24, 25]. Separate models were run for the composite outcome score and each individual outcome measure, controlling for demographic variables. For our analyses, we followed similar studies that have interpreted the Diabetes Care Profile Control Problems scale outcome as a continuous variable [22, 23, 26]. While the outcome variables could be interpreted as ordinal variables, we tested the distribution and have found they are relatively normally distributed. Sensitivity analysis between ordinal and linear regressions indicated similar results thus a linear regression was maintained. All analyses were performed using SPSS v.29.0.0.0 (SPSS Inc., Chicago, IL, USA).

## Results

### Sample Characteristics

The study sample comprised 1,225 Black/African American men with T2D. Participants averaged 41.9 years of age (SD = 14.5) and reported a mean Body Mass Index of 31.0 (SD = 9.2). The majority resided in urban areas (52.4%, n = 642), with others living in suburban (36.1%, n = 442) and rural areas (11.1%, n = 136). Most participants were married or partnered (61.1%, n = 749), while others reported never being married (27.6%, n = 338), being divorced or separated (8.8%, n = 108), or widowed (2.5%, n = 31). Regarding employment, the majority were employed (78.2%, n = 958), with others identifying as retired (9.7%, n = 119), not employed (5.9%, n = 72), disabled (4.4%, n = 54), or students (1.9%, n = 23). Annual household income showed considerable variation, with 37.8% (n = 463) reporting income under $50,000, including 11.4% (n = 140) earning less than $24,999 and 26.4% (n = 323) earning between $25,000-$49,999. Participants reported an average of 2.5 chronic conditions (SD = 1.9) in addition to their T2D diagnosis. Complete demographic information is presented in Table 1.

**Table 1:** Sample Characteristics of Black/African American Men With Type 2 Diabetes (N = 1,225)

Characteristic	Mean (SD) Or n (%)
Age (years)	41.9 (± 14.5)
Body Mass Index (kg/m^2^)	31.0 (± 9.2)
Number of Chronic Conditions	2.5 (± 1.9)
Residential Area	
Urban	642 (52.4%)
Suburban	442 (36.1%)
Rural	136 (11.1%)
Other	4 (0.3%)
Educational Attainment	
Some high school, no diploma	20 (1.6%)
High school diploma/GED	263 (21.5%)
Some college, no degree	315 (25.8%)
Technical/vocational training	43 (3.5%)
Associates degree	166 (13.6%)
Bachelor's degree	311 (25.4%)
Graduate degree	105 (8.5%)
Annual Household Income	
Less than $24,999	140 (11.4%)
$25,000-$49,999	323 (26.4%)
$50,000-$74,999	303 (24.7%)
$75,000-$99,999	223 (18.2%)
$100,000-$124,999	109 (8.9%)
$125,000-$149,999	52 (4.2%)
More than $150,000	74 (6.0%)
Marital Status	
Married/Partnered	749 (61.1%)
Never Married	338 (27.6%)
Divorced/Separated	108 (8.8%)
Widowed	31 (2.5%)
Employment Status	
Employed	958 (78.2%)
Retired	119 (9.7%)
Not Employed	72 (5.9%)
Unable to work	54 (4.4%)
Student	23 (1.9%)
Type 2 Diabetes Outcomes	
Composite Score	3.65 (2.92)
Low Blood Sugar	1.09 (0.92)
High Blood Sugar	1.21 (1.03)
Ketones	0.55 (0.92)
Severe Reactions	0.80 (1.00)

Note: SD = Standard Deviation, GED = General Educational Development program. Total percentages may not equal 100 due to rounding

### Negative T2D Outcomes

Analysis of adverse T2D outcomes revealed varying frequencies of diabetes-related complications among participants. In examining low blood sugar reactions with symptoms such as sweating, weakness, and anxiety in the past month, participants reported an average frequency of 1.09 (SD = 0.92) on the 0–4 scale. High blood sugar symptoms, including thirst, dry mouth, and fatigue, were the most commonly reported adverse outcomes in the past month (M = 1.21, SD = 1.03). The presence of ketones in urine was the least frequently reported outcome (M = 0.55, SD = 0.92). Severe low blood sugar reactions requiring assistance occurred less frequently over the past year, with a mean score of 0.80 (SD = 1.00). The composite score for negative T2D outcomes, calculated by summing all four measures (possible range 0–16), averaged 3.65 (SD = 2.92), suggesting that while participants experienced various adverse outcomes, the overall frequency remained relatively moderate.

### Social Network Composition

On average, participants reported approximately six individuals in their social networks (M = 5.8, SD = 4.3) with moderate relationship heterogeneity (M = 0.8, SD = 0.4). Networks contained the highest proportion of friends (18.8%), followed by healthcare providers (17.7%), parents (15.1%), siblings (12.0%), and spouses (11.7%). Extended family members (10.6%) and children (4.9%) comprised smaller proportions of participants' networks. On average, 18.6% of network members also had T2D. Participants reported speaking to network members 3–5 days a week (M = 3.2, SD = 1.0) and speaking to them about T2D 1–2 days a week (M = 2.4, SD = 1.2). A majority of network members were perceived as very supportive (64.8%), with participants reporting high levels of overall social support (M = 3.6, SD = 0.6). Participants perceived network members to eat healthy and be physically active sometimes (M = 2.3, SD = 0.5; M = 2.2, SD = 0.6; respectively).

### Composite Negative T2D Outcomes Model

Examining network interaction patterns, diabetes-specific discussions were positively associated with negative outcomes (β = 0.329, p < 0.001), while general communication frequency showed a negative association (β = −0.221, p < 0.001). Network members characterized as very supportive were associated with negative outcomes (β = −0.209, p < 0.001), and having infrequent contact with network members was negatively associated with outcomes (β = −0.136, p = 0.002). Network composition analysis revealed that higher percentages of siblings (β = 0.089, p = 0.034), extended family members (β = 0.105, p = 0.012), and children (β = 0.087, p = 0.015) in one's network were positively associated with negative outcomes. Mean physical activity frequency among network members showed a negative association with negative outcomes (β = −0.093, p = 0.008). See Table 2 for full model results.

**Table 2:** Social Network Characteristics Predicting Negative T2D Outcomes

Network Characteristics	Composite Score	Low Blood Sugar	High Blood Sugar	Ketones	Severe Reactions
Model R^2^	0.254	0.188	0.104	0.163	0.178
Network Composition					
Percent spouse	0.079 (0.082)	0.051 (0.276)	0.084 (0.091)	0.098* (0.041)	0.000 (0.995)
Percent child	0.087* (0.015)	0.026 (0.490)	0.110** (0.005)	0.075* (0.047)	0.075* (0.047)
Percent parent	0.010 (0.845)	−0.024 (0.638)	0.038 (0.486)	0.042 (0.427)	0.042 (0.427)
Percent friend	0.085 (0.112)	0.005 (0.926)	0.078 (0.183)	0.120* (0.033)	0.040 (0.474)
Percent sibling	0.089* (0.034)	0.036 (0.411)	0.109* (0.018)	0.073 (0.099)	0.073 (0.099)
Percent extended family	0.105* (0.012)	0.060* (0.012)	0.111* (0.015)	0.095* (0.030)	0.040 (0.354)
Percent healthcare provider	0.106 (0.051)	−0.022 (0.691)	0.120* (0.043)	0.168** (0.003)	0.042 (0.460)
Percent with T2D	0.036 (0.202)	0.020 (0.486)	0.035 (0.257)	0.052 (0.082)	0.007 (0.817)
Network Structure					
Network size	−0.014 (0.586)	−0.011 (0.688)	−0.044 (0.128)	0.010 (0.714)	0.003 (0.924)
Relationship heterogeneity	0.037 (0.168)	0.032 (0.242)	0.019 (0.518)	0.038 (0.180)	0.024 (0.394)
Network Interaction					
Percent talk < 1x/week	−0.136** (0.002)	−0.115* (0.013)	−0.083 (0.088)	−0.075 (0.112)	−0.133** (0.004)
Mean talk frequency	−0.221*** (< 0.001)	−0.219*** (< 0.001)	−0.149* (0.010)	−0.132* (0.017)	−0.161** (0.003)
Mean T2D talk frequency	0.329*** (< 0.001)	0.232*** (< 0.001)	0.195*** (< 0.001)	0.282*** (< 0.001)	0.285*** (< 0.001)
Mean perception of network PA frequency	−0.093** (0.008)	−0.102** (0.005)	−0.045 (0.245)	−0.093* (0.012)	−0.038 (0.298)
Mean perception of network healthy eating frequency	−0.004 (0.906)	0.008 (0.838)	−0.037 (0.361)	0.000 (0.991)	0.015 (0.692)
Percent very supportive	−0.209*** (< 0.001)	−0.152* (0.018)	−0.137* (0.043)	−0.166* (0.011)	−0.173** (0.008)
Mean support	0.071 (0.250)	0.103 (0.110)	0.027 (0.693)	0.022 (0.731)	0.055 (0.393)

Note: Values represent standardized regression coefficients (β) with p-values in parentheses. T2D: Type 2 Diabetes; PA: Physical Activity. All models control for age, education, residential area, employment status, income, relationship status, and body mass index. *p < 0.05, **p < 0.01, ***p < 0.001

### Low Blood Sugar Reactions (Past Month) Mode

For interaction patterns, diabetes-specific discussions showed a positive association (β = 0.232, p < 0.001), while mean general communication frequency showed a negative association (β = −0.219, p < 0.001). Having very supportive network members was associated with fewer low blood sugar reactions (β = −0.152, p = 0.018). In terms of network health behaviors, physical activity among network members showed a negative association (β = −0.102, p = 0.005). For network composition, extended family (β = 0.060, p = 0.012) and sibling presence (β = 0.089, p = 0.034) were positively associated with low blood sugar reactions. See Table 2 for full model results.

### High Blood Sugar Symptoms Model

Communication analysis showed diabetes-specific discussions were positively associated with symptoms (β = 0.195, p < 0.001), while mean communication frequency showed a negative association (β = −0.149, p = 0.010). Network composition variables were significantly associated with symptoms, including percentage of children (β = 0.110, p = 0.005), siblings (β = 0.109, p = 0.018), extended family (β = 0.111, p = 0.015), and healthcare providers (β = 0.120, p = 0.043). Having very supportive network members was negatively associated with symptoms (β = −0.137, p = 0.043), while higher frequency of diabetes-specific discussions showed a positive association (β = 0.195, p < 0.001). See Table 2 for full model results.

### Ketones in Urine Model

Communication patterns showed diabetes-specific discussions were positively associated with ketone presence (β = 0.282, p < 0.001), while mean communication frequency showed a negative association (β = −0.132, p = 0.017). Having very supportive network members was associated with lower ketone presence (β = −0.166, p = 0.011). Network composition analysis revealed positive associations for spouses (β = 0.098, p = 0.041), children (β = 0.075, p = 0.047), friends (β = 0.120, p = 0.033), extended family (β = 0.095, p = 0.030), and healthcare providers (β = 0.168, p = 0.003). Physical activity frequency among network members showed a negative association (β = −0.093, p = 0.012). See Table 2 for full model results.

### Severe Low Blood Sugar Reactions (Past Year) Model

Regarding communication patterns, diabetes-specific discussions were positively associated with severe reactions (β = 0.285, p < 0.001), while higher mean communication frequency (β = −0.161, p = 0.003) and infrequent contact (β = −0.133, p = 0.004) were associated with fewer severe reactions. Having very supportive network members was associated with fewer severe reactions (β = −0.173, p = 0.008). The presence of spouses (β = 0.084, p = 0.041) and children (β = 0.075, p = 0.047) in the network was positively associated with severe reactions. See Table 2 for full model results.

## Discussion

This study focused on the influence of social networks on T2D outcomes for Black/African American men, a population disproportionately affected by T2D and its complications. Key findings showed the degree of low blood sugar reactions, high blood sugar symptoms, and ketone presence were significantly associated with social network characteristics, such as communication patterns, support quality, and network composition. Additionally, while general communication and supportive network members showed significant negative associations indicating protective effects, frequent T2D-specific dialogues were continuously linked to less favorable results. These findings indicate the importance of examining social networks as a factor in managing T2D in Black/African American men to potentially reduce inequities in health outcomes [27]. Notably, participants reported relatively moderate levels of negative T2D outcomes overall. This suggests either effective disease management within this sample or potential selection bias toward individuals with better health engagement. Despite these moderate outcome levels, the significant associations observed between social network characteristics and T2D outcomes underscore the continued relevance of social factors in diabetes management even among relatively well-controlled populations. By integrating social network theory with cultural considerations, this study advances toward developing equitable, community-centered initiatives.

The consistent positive association between T2D-specific discussions and adverse outcomes was a potentially paradoxical finding. However, instead of indicating that T2D specific conversations lead to negative T2D outcomes, this finding may signify seeking help for and discussing these negative outcomes. Such conversations may reflect health and help seeking behaviors; these men may be prone to seek advice during crises [15]. For example, discussions about symptom management may arise after episodes of hypoglycemia or hyperglycemia, which indicates unresolved health challenges. Alternatively, frequent T2D talk might amplify stress or stigma, particularly if conversations focus on barriers to care and what is not accessible rather than solutions [11]. This emphasizes the significance of assessing the context, timing, and tone of health-related communications within networks. Training network members to have productive, solution-focused conversations that empower patients rather than overwhelm them should be the top priority of interventions.

Frequent general communication was linked to fewer negative outcomes among participants in this study. According to Cohen and Wills' stress-buffering hypothesis [16], routine encounters may operate as a stress buffer by encouraging emotional support and reinforcing conventional health practices. For example, by normalizing healthy routines, informal discussions about everyday activities may indirectly encourage adherence to food or medication guidelines. Beyond clinical or problem-focused contacts, the protective function of general communication emphasizes the importance of holistic social involvement in T2D care. Patients should be encouraged by healthcare professionals to keep strong social ties because of these [9].

The differential impact of support quality versus quantity emerged as a notable finding in our analysis. Having a higher percentage of "very supportive" network members was consistently associated with fewer adverse T2D outcomes across all models, while mean support levels showed no significant associations. This distinction suggests that the intensity or quality of support may be more influential than the general presence of support in managing T2D outcomes. Network members categorized as "very supportive" may provide more comprehensive assistance, ranging from emotional encouragement to practical help with medication adherence and lifestyle modifications [28]. These findings align with previous research indicating that the depth of social support, rather than its breadth, plays a crucial role in chronic disease management [11]. This pattern may be particularly relevant for Black/African American men, who often face unique cultural and structural barriers in healthcare settings [29]. The presence of deeply committed support figures may help navigate these challenges more effectively than a broader network offering more superficial support [30, 31]. These results emphasize the importance of fostering high-quality, deeply supportive relationships rather than simply expanding social networks [32].

Network composition emerged as a critical factor in T2D outcomes, with family relationships showing particularly complex associations. Higher proportions of siblings, children, and extended family members in participants' networks were linked to poorer T2D outcomes, reflecting the multifaceted nature of familial relationships where emotional closeness can coexist with competing demands and responsibilities. Familial relationships often introduce conflicting roles, such as caregiver responsibilities, financial interdependence, and cultural expectations, that can complicate T2D self-management [12]. For instance, family members may inadvertently create stress through well-intentioned but overwhelming involvement in disease management, or they may have their own health needs that require attention from the participant [33]. The presence of healthcare providers in participants' networks showed a distinct pattern, being associated with increased ketone presence, likely reflecting a reactive rather than preventive dynamic where healthcare providers become more actively involved in patients' networks as their disease severity increases [28]. This finding aligns with previous research indicating that individuals often expand their health-focused networks in response to worsening symptoms or complications [34]. These nuanced relationships underscore the importance of looking beyond simple network size to examine the quality and context of relationships within social networks, suggesting that the mere presence of family members or healthcare providers in one's network does not guarantee improved health outcomes. Instead, the nature of these relationships, including role expectations, communication patterns, and the distribution of support responsibilities, may be more critical determinants of T2D management success [35–37]. Understanding these complex dynamics is essential for developing interventions that effectively leverage existing social networks while addressing potential sources of stress and conflict.

The relationship between network health behaviors and T2D outcomes revealed an intriguing pattern that highlights the selective nature of social influence. Physical activity levels within participants' networks demonstrated a protective effect against negative T2D outcomes, supporting the concept of behavioral contagion where health practices spread through social connections [38]. This finding suggests that having physically active network members may normalize exercise behaviors and provide both direct and indirect support for maintaining physical activity routines [39]. However, perceptions of healthy eating behaviors among network members showed no significant association with T2D outcomes, indicating that dietary influences may operate through different mechanisms than physical activity. This divergence could reflect the more private nature of eating behaviors compared to physical activity, which often has a more visible and social component [14, 32]. Additionally, cultural factors specific to Black/African American communities, such as traditional food practices and social gatherings centered around meals, may moderate the influence of network members' dietary behaviors [14, 32]. The contrast between physical activity and dietary influences suggests that interventions leveraging social networks should carefully consider which health behaviors are most amenable to peer influence and may need to employ different strategies for promoting dietary changes versus physical activity engagement.

This study integrates social network theory by illuminating the complex relationships between communication patterns, social network composition, and health outcomes among Black/African American men with T2D. The integration of intersectionality [40] and the Social Ecological Model [41] provides a comprehensive framework for understanding how race, gender, and socioeconomic status intersect to shape disease management [14]. Structural barriers such as limited healthcare access and cultural norms around masculinity may amplify the stress experienced by Black/African American men, potentially exacerbating the impact of unsupportive networks [7, 29]. These intersecting challenges are particularly evident in the context of T2D-specific discussions, where men may delay seeking help until complications arise, reflecting cultural expectations of self-reliance and stoicism [7, 10, 29]. Systemic racism and economic marginalization further compound these challenges by limiting access to supportive networks and healthcare resources [42]. This study further emphasizes the need to consider both individual-level factors and broader societal influences when examining T2D management among Black/African American men. While not included in this study, future studies may find it fruitful to evaluate interaction effects between different social network composition factors as well as between social network composition and broader structural and cultural factors.

These findings have significant implications for clinical practice and intervention development. Healthcare providers should routinely assess patients' social networks to identify both sources of support and potential stressors, incorporating this information into personalized care plans [30, 31]. Health programs could leverage existing networks by training family members in effective communication strategies and incorporating peer mentors who share cultural backgrounds and experiences [15]. Programs should be culturally tailored to align with Black/African American men's preferences and values by potentially framing self-care and discussions about challenges as an expression of strength and leadership rather than weakness [43–45]. This might include emphasizing how effective T2D management enables men to fulfill valued social roles and responsibilities [46]. Electronic health records could be enhanced to include social network mapping, enabling providers to track changes in support systems over time and adjust care strategies accordingly. Such comprehensive, culturally competent approaches that acknowledge both social network dynamics and cultural context may be more effective in promoting positive health outcomes among Black/African American men with T2D.

### Limitations

This study has several limitations. Its cross-sectional design prohibits causal inferences, and self-reported data may introduce recall bias. Online recruitment, while efficient, may exclude individuals who need help equally due to limited digital access, potentially skewing socioeconomic representation. Additionally, the use of broad network categories (e.g., “extended family”) may overlook discreet relationship dynamics. Future studies should employ longitudinal designs and mixed methods approaches to explore how networks evolve with disease progression. Further investigation into the mechanisms linking support quality to physiological outcomes (e.g., stress biomarkers) is also justified. One limitation of our analytic approach is that we treated the summed scores of ordinal items as continuous variables in linear regression models. While the distribution of these scores met standard criteria for normality, the underlying data were originally ordinal. This approach, although widely used and empirically justified by our sensitivity analyses, may not fully account for the potential non-linear relationships between the variables. Additionally, participants in this study reported relatively low levels of negative T2D outcomes overall, which may indicate successful disease management within this sample or reflect potential selection bias toward individuals with better health status or greater health engagement. This finding warrants caution in generalizing results to the broader population of Black/African American men with T2D, particularly those experiencing more severe complications or limited healthcare access. The relatively controlled T2D status among participants may have influenced the observed associations between social network characteristics and outcomes, potentially underestimating the impact of social factors among men with more poorly managed diabetes. Finally, culturally adaptive approaches should be developed in partnership with Black/African American communities to ensure relevance and sustainability.

## Conclusion

This study highlights the dual role of social networks as both protective and risk factors in T2D management among Black/African American men. By elucidating the relationship between communication patterns, support quality, and cultural context, it provides a roadmap for reducing disparities through community-engaged, intersectional approaches. Addressing T2D in this population requires moving beyond individual-level interventions to reshape the social ecosystems that profoundly influence health outcomes.
